# Dietary Nicotinamide Mononucleotide, a Key NAD^+^ Intermediate, Alleviates Body Fat Mass and Hypertriglyceridemia by Enhancing Energy Expenditure with Promotion of Fat Oxidation and Hepatic Lipolysis and Suppressing Hepatic Lipogenesis in *db/db* Mice

**DOI:** 10.3390/metabo15050333

**Published:** 2025-05-18

**Authors:** Bungo Shirouchi, Sarasa Mitsuta, Mina Higuchi, Mai Okumura, Kazunari Tanaka

**Affiliations:** 1 Department of Nutrition Science, Faculty of Nursing and Nutrition, University of Nagasaki, Siebold, 1-1-1 Manabino, Nagayo-cho, Nishi-Sonogi-gun, Nagasaki 851-2195, Japan; 2 Nutrition Science Course, Division of Human Health Science, Graduate School of Regional Design and Creation, University of Nagasaki, Siebold, 1-1-1 Manabino, Nagayo-cho, Nishi-Sonogi-gun, Nagasaki 851-2195, Japan; 3 Regional Partnership Center, University of Nagasaki, Siebold, 1-1-1 Manabino, Nagayo-cho, Nishi-Sonogi-gun, Nagasaki 851-2195, Japan

**Keywords:** NMN, obesity, dyslipidemia, respiratory gas analysis, metabolic profile, hepatic fatty acid metabolism, adiponectin, T-cadherin

## Abstract

**Background/Objectives**: Supplementation with nicotinamide mononucleotide (NMN), a key nicotinamide adenine dinucleotide (NAD^+^) intermediate, exerts anti-aging, anti-obesity, and anti-diabetic effects in animal experiments. However, previous studies have evaluated NMN supplementation using oral administration in drinking water or by intraperitoneal administration. No studies have reported whether NMN exerts beneficial effects when incorporated into the diet. The diet is a multicomponent mixture of many nutrients that may interact with each other, thus weakening the effects of NMN. In the present study, we evaluated whether dietary NMN intake protects obese diabetic *db/db* mice from obesity-related metabolic disorders, such as dyslipidemia, hepatic steatosis, hyperglycemia, and hyperinsulinemia. **Methods**: Five-week-old male *db/db* mice were randomly assigned to two groups and fed for four weeks either a control diet containing 7% corn oil and 0.1% cholesterol (CON group, *n* = 6) or a diet supplemented with 0.5% NMN (NMN group, *n* = 5). **Results**: After 4 weeks of feeding, dietary NMN intake alleviated obesity, hypertriglyceridemia, and hepatic triglyceride accumulation in *db/db* mice. Respiratory gas analysis indicated that dietary NMN intake significantly enhanced energy expenditure by suppressing carbohydrate oxidation and increasing fat oxidation after 3 weeks of feeding. Additionally, the suppression of the increase in plasma triglyceride (TG) levels by dietary NMN intake was attributable to a reduction in hepatic TG levels through the suppression of fatty acid synthesis and the enhancement of fatty acid β-oxidation in the liver. Furthermore, the improvement in hepatic fatty acid metabolism induced by dietary NMN intake was partially responsible for the significant increase in plasma adiponectin and soluble T-cadherin levels. **Conclusions**: This is the first report to show that dietary NMN intake but not oral administration in drinking water or intraperitoneal administration alleviates body fat mass and hypertriglyceridemia by enhancing energy expenditure, with preferential promotion of fat oxidation, the enhancement of hepatic lipolysis, and the suppression of hepatic lipogenesis in *db/db* mice.

## 1. Introduction

Metabolic syndrome is a cluster of metabolic abnormalities, including abdominal obesity, dyslipidemia, impaired fasting glucose, and high blood pressure, that can lead to the development and progression of diabetes and cardiovascular diseases [1]. According to the World Health Organization report on the global prevalence of overweight and obesity in 2022, 2.5 billion adults over the age of 18 years have a body mass index (BMI) of 25 or higher, of which more than 890 million will have a BMI of 30 or higher [2]. This corresponds to 43% of adults aged 18 years and older being overweight (43% of men and 44% of women), representing a major increase from 25% of adults in 1990 [2]. Obesity, particularly abdominal fat accumulation, causes metabolic syndrome; therefore, attaining and maintaining a healthy body weight is critical. Abdominal fat is mainly derived from dietary fat or excess energy intake because of an imbalance between energy intake and expenditure. Therefore, to combat the overweight and obesity epidemics, it is important to reduce energy intake and increase energy expenditure. To this end, several food ingredients have been extensively studied and reported to exert anti-obesity effects by enhancing energy expenditure, including 10trans, 12cis-conjugated linoleic acid [3], campestenone [4], capsinoids (capsaicin analogs) [5], fish oil [6], pterostilbene [7], and the probiotic *Lactobacillus gasseri* SBT2055 [8].

Nicotinamide adenine dinucleotide (NAD^+^) is a well-known coenzyme for hydride-transfer enzymes and a substrate for NAD^+^-consuming enzymes, including poly (ADP-ribose) polymerases (PARPs), sirtuins, CD38/CD157, sterile alpha, and toll/interleukin-1 receptor motif-containing 1 (SARM1) [9]. A review of studies on NAD^+^ metabolism [9] showed that NAD^+^ levels in the body change depending on the environment and nutritional state and that NAD^+^ functions as an energy sensor, playing an important role in a wide variety of biological phenomena, including metabolism, inflammation, differentiation, and aging. Several studies using rodents and humans have indicated that the NAD^+^ content declines with age in various organs and tissues, such as the pancreas, adipose tissue, skeletal muscle, liver, skin, and brain [10,11,12,13,14,15]. Nicotinamide mononucleotide (NMN) is known as a biosynthetic precursor of NAD^+^ and is synthesized from nicotinamide and 5-phosphoribosyl-1-pyrophosphate by nicotinamide phosphoribosyltransferase (NAMPT) [9]. Once synthesized, NMN is converted to NAD^+^ by one of three NMN adenylyltransferases (NMNAT1–3) [9]. Therefore, enhancing NAD^+^ biosynthesis by administering NMN is expected to provide significant preventive effects against various diseases by replenishing the intracellular NAD^+^ pool. Previous studies using animal models [16,17,18,19] have reported that NMN administration suppresses body weight gain, improves glucose tolerance and mitochondrial function [16], protects against inflammation [17], and improves cognitive function [18,19]. It has also been shown that administration of NMN (100 and 300 mg/kg/day) to mice enhances energy metabolism [20]. The results of the abovementioned studies suggest that NMN supplementation may contribute to the alleviation and/or prevention of obesity and changes in the adipocytokine profile secreted by adipose tissue, leading to the prevention of metabolic syndrome. However, a review of studies of NMN [21], including the aforementioned studies, revealed that the methods for administering NMN to laboratory animals have been limited to intraperitoneal injections or via drinking water. To the best of our knowledge, no study has reported the effects of NMN incorporated in diet. For certain pharmaceutical ingredients, the presence of specific nutrients can reduce their absorption or effectiveness, making it important to adhere to the prescribed timing of administration—such as before meals, between meals, or after meals. The diet is a multicomponent mixture of many nutrients, and NMN, like pharmaceutical ingredients, may interact with specific nutrients, thus weakening its effects. Therefore, it is necessary to verify whether NMN has the same effect when administered in the diet as when administered via intraperitoneal injection or drinking water.

To gain insights into the physiological function of dietary NMN intake, in the present study, obese diabetic *db/db* mice, which have a lower level of energy metabolism than normal mice, such as C57BL/6J mice, were used to evaluate the effects of dietary NMN intake on the pathogenesis of obesity, energy metabolism, and lipid abnormalities.

## 2. Materials and Methods

### 2.1. Materials

NMN was provided by WELLCREATE Co., Ltd. (Nagasaki, Japan). The purity of NMN was determined using quantitative nuclear magnetic resonance at Japan Food Research Laboratories (Tokyo, Japan) and was found to be 99.8%.

### 2.2. Experimental Diets

The experimental diets were prepared according to the AIN-76 formula [22], with several modifications (composition of the control diet: corn oil content was increased from 5% to 7% and cholesterol content was 0.1%; these changes were adjusted with sucrose) (Table S1). To induce earlier-onset lipid abnormalities in experimental animals, the standard AIN-76 diet—comprising 50% sucrose, 5% corn oil, and no added cholesterol—was modified by increasing the dietary fat content (corn oil) and supplementing with 0.1% cholesterol. A previous study estimated the no-observable adverse effect level (NOAEL) for NMN to be greater than 1500 mg/kg/day [23]. In addition, Mills et al. reported that energy expenditure was increased by administering 300 mg/kg/day NMN in drinking water to mice [20]. The diet is a multicomponent mixture of many nutrients that may interact with each other, potentially weakening the effects of NMN. Based on these data, the NMN dosage in the diet used in the present study was 0.5% (Table S1).

### 2.3. Animals

All experiments were conducted in accordance with the Guidelines for Animal Experiments of University of Nagasaki, Siebold, and Law No. 105 and Notification No. 6 of the Government of Japan. The animal protocol used in this study was approved by the Institutional Review Board of University of Nagasaki, Siebold (authorization no. R04-02). Five-week-old male C57BL/6J (C57BL/6JJcl) and *db/db* (BKS.Cg-+*Lepr^db^*/+*Lepr^db^*/Jcl) mice were purchased from CLEA Japan, Inc. (Osaka, Japan). *db/db* mice exhibit hyperphagia due to a missense mutation in the leptin receptor gene and develop multiple metabolic and hormonal disorders, including hepatic lipid accumulation (i.e., metabolic-dysfunction-associated steatotic liver disease (MASLD)) and type 2 diabetes, which share many features with human metabolic syndromes [24,25]. The mice were housed individually in plastic cages in a temperature-controlled room at 22 ± 1 °C with 55 ± 5% humidity, under a 12 h light/dark cycle. After a 3-day adaptation period, the *db/db* mice were assigned to one of two groups and fed one of the following diets: control diet (CON group, *n* = 6) or NMN diet (NMN group, *n* = 5). C57BL/6J mice, the progenitors of *db/db* mice, were fed a control diet (NOR group, *n* = 6). Mills et al. evaluated energy metabolism in mice administered 300 mg/kg/day NMN in drinking water, with a sample size of *n* = 5/group [20]. Based on this previous study, the sample size in this study was set at *n* = 5–6/group. Mice in the CON and NMN groups were pair-fed diets using Rodent CAFE (KBT Oriental, Saga, Japan) and had free access to water for 4 weeks. Mice in the NOR group were allowed free access to the control diet using Rodent CAFE and had free access to water for 4 weeks. All mice were subjected to respiratory gas analysis after 3 weeks of experimental diet consumption. Feces were collected 6 days prior to the end of the experiment. Two days before anatomical examination, the rectal temperature of the mice was measured using a digital thermocouple thermometer (HD2128.1; Muromachi Kikai Co., Ltd., Tokyo, Japan). At the end of the feeding period, the body length (naso–anal) of the mice was measured using digital calipers (GDCS-150, Niigata Seiki Co., Ltd., Niigata, Japan) under isoflurane anesthesia after a 9 h fasting period. The mice were sacrificed using heart exsanguination. Blood was collected in ethylenediaminetetraacetic acid (EDTA)-2Na-containing tubes, and plasma was collected using centrifugation at 1200× *g* for 20 min at 4 °C. The liver, testes, kidneys, quadriceps femoris, abdomen (epididymal, perirenal, and mesenteric), subcutaneous white adipose tissue (WAT), and brown adipose tissue (BAT) were excised and weighed within 4.5 h. The collected samples were stored at −80 °C until further analysis. The Lee index for assessing obesity was calculated using the following formula [26]:

$$\mathrm{Lee}\;\mathrm{index}=\sqrt[{3}]{body\;weight\;\left(g\right)}\times 10³/naso-anal\;length\;(cm).$$

**Figure 1:**
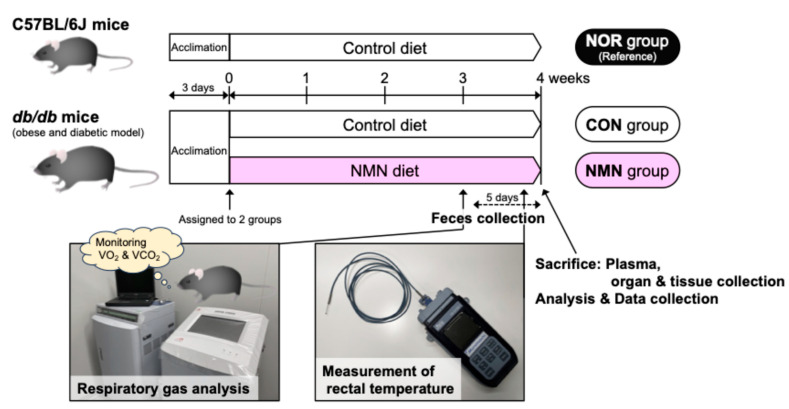
Outline of the experimental design for this study.

The experimental design of this study is outlined in Figure 1.

### 2.4. Respiratory Gas Analysis

After 3 weeks of feeding the experimental diets, each mouse was placed in an acrylic metabolic chamber (120 × 150 × 240 mm; ARCO SYSTEM, Inc., Chiba, Japan) for 24 h to measure VO_2_ (oxygen exhaustion) and VCO_2_ (carbon dioxide emission) as described previously [27]. During respiratory gas analysis, mice in the CON and NMN groups were pair-fed and had free access to water. Carbohydrate and fat oxidation and energy expenditure were calculated as previously described [27].

### 2.5. Measurement of Plasma Biochemical Parameters

Plasma levels of triglycerides (TGs), total cholesterol (T-Chol), phospholipids (PLs), glucose, alanine transaminase (ALT), and cholinesterase (ChE) were measured using commercial enzyme assay kits (FUJIFILM Wako Pure Chemical Co., Osaka, Japan). Plasma albumin levels were measured using a commercial assay kit based on the bromocresol green method (FUJIFILM Wako Pure Chemical Co.) [28]. TG and Chol levels in the plasma high-density lipoprotein (HDL) fraction were measured using commercial enzyme kits. Plasma levels of non-HDL-TG were calculated as the difference between TG and HDL-TG levels. Plasma non-HDL cholesterol levels were calculated as the difference between T-Chol and HDL cholesterol levels. Plasma levels of adiponectin, insulin, and leptin were measured using commercial mouse enzyme-linked immunosorbent assay (ELISA) kits (mouse/rat adiponectin ELISA kit, Otsuka Pharmaceutical, Tokyo, Japan; LBIS mouse insulin ELISA kit (U-type and T-type), Shibayagi, Gunma, Japan; Mouse/Rat Leptin ELISA kit, Morinaga Institute of Biological Science, Inc., Kanagawa, Japan). Plasma T-cadherin levels were measured using commercial human ELISA kits (human T-cadherin (130 kDa) ELISA kit, and human T-cadherin (100 kDa + 130 kDa) ELISA kits; Immuno-Biological Laboratories Co., Ltd., Gunma, Japan). The human ELISA kits were also cross-reactive with mouse plasma samples. The plasma levels of T-cadherin (100 kDa) were calculated as the difference between the levels of the 100 kDa + 130 kDa forms and the 130 kDa form.

### 2.6. Measurement of Hepatic Contents of Lipids, Glycogen, NAD^+^, and NADH

Total lipids were extracted from the liver (0.1 g) as previously described [27]. Hepatic TG, Chol, PL, and glycogen contents were measured as previously described [27]. Hepatic levels of NAD^+^ and nicotinamide adenine dinucleotide hydride (NADH) were measured using a commercial enzyme assay kit (NAD/NADH Assay Kit-WST; DOJINDO LABORATORIES, Kumamoto, Japan) according to the manufacturer’s instructions.

### 2.7. Measurement of NAD^+^ and NADH Contents in Epididymal WAT

NAD^+^ and NADH contents in the epididymal WAT were measured using a commercial enzyme assay kit (NAD/NADH Assay Kit-WST) according to the manufacturer’s instructions.

### 2.8. Measurement of Adiponectin Content in Mesenteric WAT

Mesenteric WAT samples were homogenized in lysis buffer containing 100 mM Tris (pH 7.4), 150 mM NaCl, 1 mM ethylene glycol bis(2-aminoethylether)-N,N,N’,N’-tetraacetic acid, 1 mM EDTA, 1% Triton X-100, 0.5% sodium deoxycholate, 1 mM phenylmethylsulfonyl fluoride, 1× phosphatase inhibitor cocktail (PhosSTOP™; Roche Diagnostics K.K., Tokyo, Japan), and 1× protease inhibitor cocktails (cOmplete™; Roche Diagnostics K.K.). Homogenates were incubated for 2 h at 4 °C and then centrifuged at 16,440× *g* for 20 min at 4 °C. The adiponectin content in the homogenate supernatants of mesenteric WAT was determined using a commercial mouse ELISA kit (mouse/rat adiponectin ELISA kit). The total protein concentration was determined using the DC™ protein assay (BioRad Laboratories, Inc., Tokyo, Japan).

### 2.9. Measurement of Hepatic Fatty Acid Metabolism-Related Enzyme Activities

Frozen livers (0.5 g) were homogenized in 12 volumes of a 0.25 M sucrose solution containing 1 mM EDTA in 10 mM Tris-HCl buffer (pH 7.4). Hepatic subcellular fractionation was performed using centrifugation, as previously described [29]. The enzymatic activities of fatty acid synthase (FAS) in the cytosolic fraction and carnitine palmitoyltransferase (CPT) in the mitochondrial fraction were measured, as previously described [29].

### 2.10. Measurement of mRNA Levels in Liver and Epididymal White Adipose Tissues

Total RNA was extracted from 0.1 g of liver and epididymal WAT samples that had been soaked in RNA Save (Biological Industries Israel Beit Haemek Ltd., Haemek, Israel), and it was then converted to cDNA as previously described [27]. In the liver, mRNA levels of genes involved in fatty acid metabolism, such as *Acaca*, encoding acetyl-CoA carboxylase 1; *Fasn*, encoding FAS; *Cpt1a*, encoding CPT1; *Cpt2*, encoding CPT2; *Srebf1*, encoding sterol regulatory element binding protein 1; and *Nr1h3*, encoding liver X receptor alpha (LXRα); those involved in NAD^+^ metabolism, such as *Nmnat1*, encoding nicotinamide mononucleotide adenylyltransferase 1 (NMNAT1), and *Sirt1*, encoding NAD^+^-dependent protein deacetylase sirtuin-1 (SIRT1); and those involved in adiponectin signaling, such as *Adipor1*, encoding adiponectin receptor (AdipoR)1, and *Adipor2*, encoding AdipoR2, were measured. In epididymal WAT, the mRNA levels of genes involved in fatty acid synthesis, such as *Fasn*; adipocytokines, such as *Adipoq,* encoding adiponectin; genes involved in thermogenesis, such as *Ucp2*, encoding uncoupling protein 2; *Adrb3*, encoding beta-3 adrenergic receptor; and *Prdm16*, encoding PR domain-containing 16, were also measured. Several polymerase chain reaction (PCR) amplifications were also performed with SYBR Green (THUNDERBIRD^®^ SYBR^®^ qPCR Mix; Toyobo Co., Ltd., Osaka, Japan), as previously described [27]. The primer sequences used in this study are listed in Table S2. In addition, PCR amplifications were performed using TaqMan probes (THUNDERBIRD^®^ Next Probe qPCR Mix (Toyobo Co., Ltd.) and Assay-on-Demand, Gene Expression Products (*Rn18s*; Mm04277571_s1, *Srebf1*; Mm00550338_m1, *Adipoq*; Mm04933656_m1, *Prdm16*; Mm00712556_m1, from Thermo Fisher Scientific Inc., Pleasanton, CA, USA)). Stable internal reference genes are crucial for quantitative reverse-transcription PCR. In the present study, five housekeeping genes (*Rpl32*, *Rpl13a*, *Gapd*, *Pgk1*, and *Hprt1*) in the liver and three housekeeping genes (*Rn18s*, *Rpl32*, and *Tbp*) in epididymal WAT were evaluated. BestKeeper calculates the stability of the candidate housekeeping genes based on the standard deviation of their quantification cycle (Cq) values [30]. Based on these results, *Rpl32* in the liver and *Rn18s* in epididymal WAT were selected as the most robust housekeeping genes (Figure S1). Relative mRNA levels were determined using the Pfaffl method [31].

### 2.11. Statistical Analysis

All data were included in the statistical analysis, with no exclusions made in this study. All values are expressed as the mean ± standard error of the mean. Data from the NOR group were treated as reference data and were not used for statistical analysis. The *F*-test was used to assess the equality of variance between the CON and NMN groups. Statistical analyses of parametric data with equal or unequal variances were performed using the Student’s *t*-test or Welch’s *t*-test, respectively. Pearson’s correlation coefficient was calculated, and linear regression analysis was performed using EZR (version 1.68), a graphical user interface of R (The R Foundation for Statistical Computing, Vienna, Austria) [32]. Results with *p* < 0.05 were considered statistically significant, and those with 0.05 ≤ *p* < 0.1 were considered a tendency.

## 3. Results

### 3.1. Effects of Dietary NMN Intake on Nutrients Oxidation in Obese Diabetic db/db Mice

**Figure 2:**
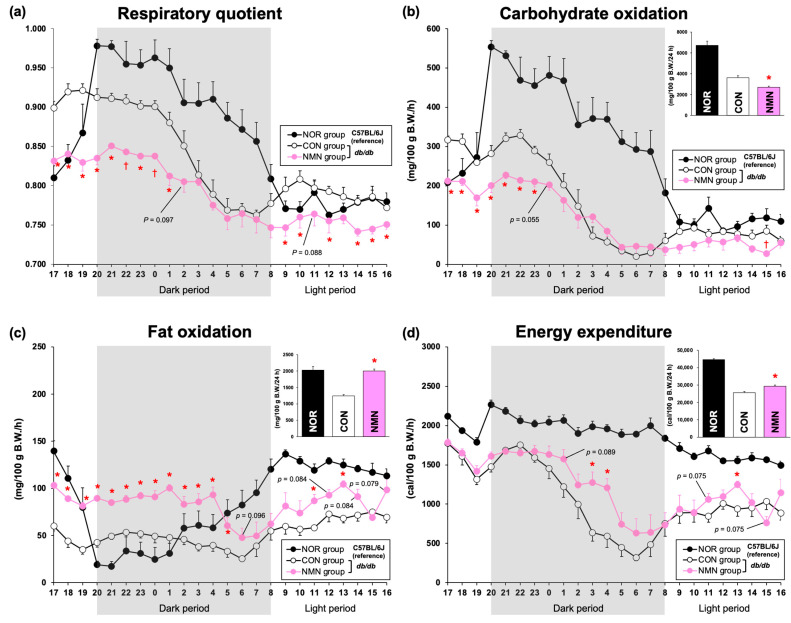
Effects of dietary NMN intake on energy metabolism in obese diabetic *db/db* mice. Time course of the changes in (**a**) respiratory quotient (RQ), (**b**) carbohydrate oxidation, (**c**) fat oxidation, and (**d**) energy expenditure in NOR, CON, and NMN groups fed the experimental diets for 3 weeks. The insets represent the amounts of carbohydrate oxidation, fat oxidation, and energy expenditure per day. Data are expressed as the mean ± standard error of the mean (NOR and CON groups, *n* = 6/group; NMN group, *n* = 5/group). * *p* < 0.05 (vs. CON group) analyzed using Student’s *t*-test. ^†^ *p* < 0.05 (vs. CON group) analyzed using Welch’s *t*-test.

To examine the effects of dietary NMN intake on nutrient oxidation, respiratory gas analysis was performed on mice after 3 weeks of feeding. Because food intake affects energy expenditure, mice in the CON and NMN groups were fed a limited amount of the experimental diets (NOR group, 2.25 ± 0.09 g; CON group, 3.50 ± 0.04 g; NMN group, 3.50 ± 0.07 g) during the analysis in the metabolic chambers. The respiratory quotient (RQ) remained consistently lower in the NMN group than in the CON group (Figure 2a). In particular, the RQ of the NMN group was significantly lower than that of the CON group during the dark period from 8 p.m. to 1 a.m. (active period) and the light periods from 9 a.m., 10 a.m., 12 a.m., and 2 p.m. to 7 p.m. (inactive period) (Figure 2a). Carbohydrate oxidation was significantly lower in the NMN group than the CON group during the dark period from 8 p.m. to 11 p.m. and the light periods from 3 p.m. and 5 p.m. to 7 p.m. (Figure 2b). Total carbohydrate oxidation was significantly lower in the NMN group (Figure 2b). In contrast, fat oxidation was consistently higher in the NMN group than the CON group (Figure 2c). Fat oxidation was significantly higher in the NMN group than the CON group during the dark period from 8 p.m. to 5 a.m. and the light periods from 11 a.m., 1 p.m., and 5 p.m. to 7 p.m., leading to significantly higher levels of total fat oxidation (Figure 2c). The energy expenditure of the NMN group was significantly higher than that of the CON group during the dark period (3 a.m. to 4 a.m.) and the light period (1 p.m.), leading to significantly higher total energy expenditure (Figure 2d). Therefore, dietary NMN intake enhances energy expenditure by suppressing carbohydrate oxidation and increasing fat oxidation (Figure 2d).

### 3.2. Effects of Dietary NMN Intake on Morphometric Variables in Obese Diabetic db/db Mice

Table 1 summarizes the morphometric variables of the mice after the 4-week feeding period. No significant differences were observed in food intake, collected fecal weight, or organ or tissue weights, including the spleen, testis, kidneys, quadriceps femoris, perirenal WAT, and brown adipose tissue (BAT), between the two groups. In contrast, the final body weight, body weight gain, food efficiency, body (naso–anal) length, and Lee index for assessing obesity were significantly lower in the NMN group than the CON group. The liver, abdominal WAT (especially epididymal and mesenteric WAT), and subcutaneous WAT weights were significantly lower in the NMN group than the CON group. Water intake and rectal temperature significantly increased in the NMN group.

### 3.3. Effects of Dietary NMN Intake on Biochemical Parameters in Plasma, the Liver, Epididymal WAT, and Mesenteric WAT of Obese Diabetic db/db Mice

Table 2 summarizes the biochemical parameters of the plasma, liver, epididymal WAT, and mesenteric WAT of obese diabetic *db/db* mice after a 4-week feeding period. No significant differences were observed in the plasma levels of T-Chol, non-HDL Chol, PLs, glucose, insulin, or leptin between the two groups. The plasma HDL Chol levels tended to be higher in the NMN group than the CON group. The plasma levels of adiponectin, T-cadherin, ALT, and ChE were significantly higher in the NMN group than the CON group, while the plasma albumin level was significantly lower in the NMN group. The *db/db* mice in the CON group exhibited obesity, hypertriglyceridemia, and hepatic TG accumulation. However, in addition to improving obesity, dietary NMN intake significantly decreased plasma TG levels by suppressing hepatic TG accumulation. Hepatic PL and glycogen content tended to be lower in the NMN group than the CON group. Hepatic NAD^+^ and NADH levels were markedly increased in the NMN group compared to those in the CON group. In contrast, NAD^+^ and NADH levels in epididymal WAT were significantly lower in the NMN group. Additionally, the adiponectin content in mesenteric WAT did not differ between the two groups. These results were reflected in the reduction in epididymal and mesenteric WAT weights after dietary NMN intake.

**Figure 3:**
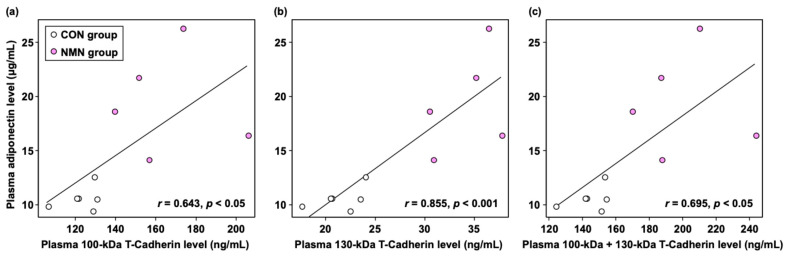
Correlations between adiponectin levels and (**a**) 100 kDa T-cadherin levels, (**b**) 130 kDa T-cadherin levels, and (**c**) 100 kDa + 130 kDa T-cadherin levels in the plasma of *db/db* mice. Individual values are shown (*n* = 11).

To further investigate whether there was a relationship between adiponectin levels and the levels of each form of soluble T-cadherin (100 kDa, 130 kDa, and 100 kDa + 130 kDa) in mouse plasma, as is the case in human plasma, we conducted a single regression analysis. As shown in Figure 3, plasma adiponectin levels were significantly and positively correlated with the plasma levels of 100 kDa T-cadherin (*r* = 0.643, *p* < 0.05), 130 kDa T-cadherin (*r* = 0.855, *p* < 0.001), and 100 kDa + 130 kDa T-cadherin (*r* = 0.695, *p* < 0.05) in *db/db* mice.

### 3.4. Effects of Dietary NMN Intake on Activities of Hepatic Enzymes Related to Fatty Acid Metabolism in Obese Diabetic db/db Mice

**Figure 4:**
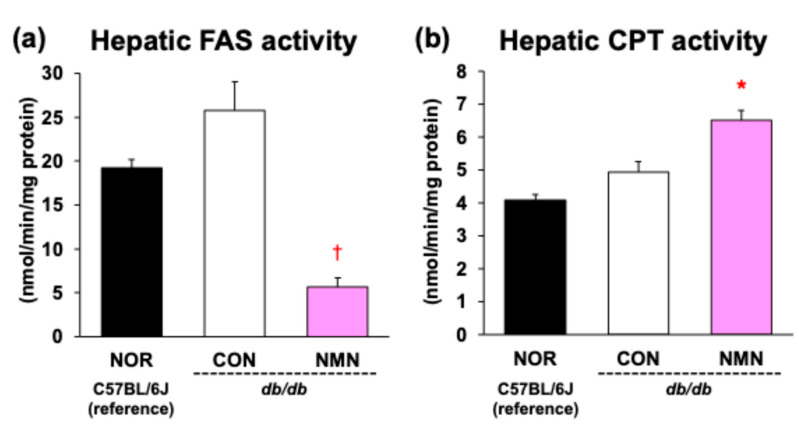
Effects of dietary NMN intake on (**a**) FAS and (**b**) CPT activities in the liver of obese diabetic *db/db* mice. Data are expressed as the mean ± standard error of the mean (NOR and CON groups, *n* = 6/group; NMN group, *n* = 5/group). * *p* < 0.05 (vs. CON group) analyzed using Student’s *t*-test. ^†^ *p* < 0.05 (vs. CON group) analyzed using Welch’s *t*-test.

Figure 4 shows enzyme activities in the livers of *db/db* mice after a 4-week feeding period. The activity of FAS, a key enzyme in fatty acid synthesis, was significantly suppressed in the NMN group compared to the CON group. In contrast, the activity of CPT, a rate-limiting enzyme of fatty acid β-oxidation, was significantly enhanced by dietary NMN intake.

### 3.5. Effects of Dietary NMN Intake on mRNA Levels in the Liver and Epididymal WAT of Obese Diabetic db/db Mice

Table 3 summarizes the effects of dietary NMN intake on the expression of several genes related to fatty acid synthesis, fatty acid β-oxidation, NAD^+^ metabolism, and adiponectin signaling in the liver of *db/db* mice. There were no significant differences in the hepatic mRNA levels of *Nr1h3*, *Cpt1a*, *Nmnat1*, *Sirt1*, or *AdipoR1* between the two groups. *Srebf1* mRNA levels tended to be higher in the NMN group than the CON group, while *Fasn* mRNA levels tended to be lower in the NMN group. Dietary NMN intake significantly decreased *Acaca* and *AdipoR2* mRNA levels and increased *Cpt2* mRNA levels. Although we investigated the effects of dietary NMN intake on the expression of several genes related to fatty acid synthesis, adipocytokines, and thermogenesis in the epididymal WAT of *db/db* mice, no significant effects were observed.

## 4. Discussion

In the present study, we evaluated the effects of dietary NMN intake on the pathogenesis of obesity, energy metabolism, and lipid abnormalities in obese diabetic *db/db* mice, which have lower energy metabolism than C57BL/6J mice. We demonstrated that dietary NMN intake alleviates body fat mass and hypertriglyceridemia by enhancing energy expenditure, preferentially promoting fat oxidation, enhancing hepatic lipolysis, and suppressing lipogenesis in mice.

We investigated whether dietary NMN intake increases the amount of NAD^+^ and its related compounds in vivo, as reported when NMN is delivered via drinking water or intraperitoneal administration. As shown in Table 2, hepatic NAD^+^ and NADH levels were markedly increased by dietary NMN supplementation. Therefore, we evaluated the mRNA levels of the genes involved in NAD^+^ metabolism, including *Nmnat1* and *Sirt1*. However, no significant differences were observed in the expression levels of these genes (Table 3). Further studies are needed to evaluate whether dietary NMN intake changes the expression levels and activities of proteins involved NAD^+^ metabolism. Our results suggest that the increase in NAD^+^ and its metabolite content in the liver was due to increased substrate (NMN) levels associated with dietary NMN intake. In contrast, no significant increase in the NAD^+^ or NADH content was observed in epididymal WAT, and this was accompanied by a significant decrease in epididymal WAT weight due to dietary NMN intake (Table 2). Unlike intraperitoneal NMN administration, dietary NMN intake increased NAD^+^ levels only in the liver, which supports the results of previous studies [20,33,34]. Mills et al. showed that oral administration of NMN via drinking water to mice tended to increase hepatic NAD^+^ content in a dose-dependent manner, but no significant increase was observed in NAD^+^ content in other tissues, such as skeletal muscle or WAT [20]. In a previous study in which chronic cardiotoxicity was induced in C57BL/6 mice using intraperitoneal injection of doxorubicin for 5 d, 500 mg/kg/d of NMN in drinking water provided ad libitum for 60 d resulted in a relative increase in NAD^+^ levels in the blood but not in the heart [33]. Furthermore, in a previous study in which male C57BL/6 mice aged 6–8 weeks were orally administered 500 mg/kg NMN, the NAD^+^ content in the liver and kidney significantly increased 6 h after administration, while the content in other tissues (brain, heart, lung, and muscle) remained unchanged [34]. In contrast, in the previous study, in which C57BL/6 mice with acute cardiotoxicity induced by a single intraperitoneal injection of doxorubicin were intraperitoneally administered 180 mg/kg/d of NMN for 5 d before and after doxorubicin injection, the relative NAD^+^ levels in the blood and heart increased [33]. The aforementioned findings, observed with the intraperitoneal administration of NMN (i.e., increased NAD^+^ levels in extrahepatic tissues), differ from those obtained with oral administration in previous studies or with dietary supplementation, as reported in our study. Taken together, these data, including our results, suggest that orally administered NMN is transported through the portal vein and then largely utilized by the liver, whereas intraperitoneally injected NMN reaches more extrahepatic tissues than orally administered NMN and increases the NAD^+^ content in these tissues. In other words, orally administered NMN, unlike intraperitoneally injected NMN, has a greater first-pass effect in the liver, suggesting that the amount transferred to extrahepatic tissues, such as WAT, is reduced.

Because obesity, especially abdominal fat deposition, can trigger metabolic syndrome, it is critical to maintain a healthy body weight and prevent fat deposition. Uddin et al. reported that intraperitoneal injection of 500 mg/kg/d NMN for 18 d in high-fat-diet-fed offspring of obese mothers (mice) reduced adiposity and improved glucose tolerance and mitochondrial function [16]. In addition, Mills et al. reported that the administration of 300 mg/kg/day NMN to mice in drinking water for 12 months suppressed age-associated body weight gain and increased food and water intake [20]. Except for food intake, these behaviors were consistent with our results in *db/db* mice fed a diet supplemented with NMN (Table 1). Compared with the NMN group, *db/db* mice in the CON group had a higher Lee index value, indicating that they were obese (Table 1). Autopsy findings showed that dietary NMN significantly reduced the abdominal and subcutaneous WAT weights, similar to the effects of orally administered NMN in drinking water, confirming the anti-obesity effect of dietary NMN intake (Table 1). Thus, our results are consistent with the observations of previous studies [16,20], and this is the first report of the anti-obesity effect of dietary NMN intake. Several studies have shown that functional food components exert anti-obesity effects by altering energy metabolism [3,4,5,6,7,8]. A previous study by Mills et al. also reported that administering 100 and 300 mg/kg/day of NMN in drinking water to mice increased their energy expenditure; however, it was unclear whether the increased energy expenditure was accompanied by changes in the metabolic profile of nutrients (carbohydrates and fats) [20]. Niu et al. reported that access to drinking water with 500 mg/L (*w*/*v*) NMN for 40 d significantly increased the water intake of C57BL/6 mice on day 21 and heat production after 40 d [35]. Furthermore, Yamaguchi et al. reported that administering 500–1000 mg/kg/day of NMN to adipocyte-specific *Nampt*-knockout (ANKO) mice for 8 weeks resulted in greater cold tolerance than that of ANKO mice without NMN administration and that NMN administration restored heat production in ANKO mice [36]. Therefore, to understand the mechanisms underlying the anti-obesity effects of dietary NMN intake, respiratory gas analysis was performed after 3 weeks of feeding the obese diabetic *db/db* mice. As shown in Figure 2a–d, dietary NMN intake induced an increase in fat oxidation instead of carbohydrate oxidation, thereby enhancing energy expenditure. As the production of NAD^+^ from NMN by NMNATs involves the consumption of ATP (Figure S2), we hypothesized that dietary NMN preferentially selects dietary fats, which can generate more ATP than dietary carbohydrates, as a substrate for energy metabolism, leading to enhanced oxidation of fat stored in the body. Taken together, these results indicate that dietary NMN intake has an anti-obesity effect through alterations of the metabolic profiles of nutrients and the enhancement of energy expenditure in obese diabetic *db/db* mice. Additionally, the increase in water intake and rectal temperature due to dietary NMN intake was reflected in enhanced energy expenditure.

Hypertriglyceridemia has several environmental and genetic etiologies, including obesity, insulin resistance, and loss-of-function mutations that control the metabolism of TG-rich lipoproteins [37]. It is well known that hypertriglyceridemia is the most common type of lipid abnormality in patients with MASLD and metabolic-dysfunction-associated steatohepatitis [37]. In addition, hypertriglyceridemia is associated with an increased risk of atherosclerotic cardiovascular disease and pancreatitis. Lowering elevated plasma TG is important for the prevention and treatment of these diseases [37]. As shown in Table 2, dietary NMN significantly suppressed the elevated plasma TG levels in *db/db* mice. Dietary NMN intake also reduced the hepatic TG content (Table 2). To gain insights into the effects of dietary NMN on TG metabolism, the activities of hepatic enzymes related to fatty acid metabolism were analyzed. As shown in Figure 4, the activity of FAS, which is involved in de novo fatty acid synthesis in the cytosol, was markedly suppressed by dietary NMN. In contrast, the activity of CPT, which is responsible for fatty acid β-oxidation in the mitochondria, was significantly enhanced by dietary NMN intake. Hepatic mRNA levels of *Acaca* and *Fasn* were significantly decreased by dietary NMN intake, whereas *Cpt2* mRNA levels were significantly increased (Table 3), suggesting that the changes in FAS and CPT activities resulting from dietary NMN intake represent transcriptional regulation. Based on the abovementioned observations, we consider that the suppression of the increase in plasma TG levels by dietary NMN intake was due to a reduction in hepatic TG content through the suppression of fatty acid synthesis and the enhancement of fatty acid β-oxidation in obese diabetic *db/db* mice.

Adipose tissue not only stores excess energy in the form of fat but also secretes several bioactive substances known as “adipocytokines” [38]. Adiponectin is one of the most abundant secretory proteins from adipose tissue in rodents and humans, and it contributes to the control of lipid metabolism by enhancing fatty acid oxidation in the liver [39]. Activated AMPK inhibits fatty acid synthesis via acetyl-CoA carboxylase (ACC), which catalyzes the biosynthesis of malonyl-CoA from acetyl-CoA. Malonyl-CoA is a potent CPT inhibitor. Therefore, adiponectin suppresses ACC activity through AMPK signaling and consequently promotes fatty acid oxidation in the liver [39]. In the present study, dietary NMN increased the adiponectin content in mesenteric WAT by 14.5%. Although this was not statistically significant, it leads to a significant increase in plasma adiponectin levels (Table 2). Therefore, our results suggest that the enhancement of fatty acid β-oxidation in the liver by dietary NMN intake involves not only transcriptional regulation but also the coordinated regulation of the attenuated inhibition of fatty acid β-oxidation by malonyl-CoA through increased plasma adiponectin levels. T-cadherin binds to adiponectin, and it has been reported that three forms of soluble T-cadherin exist in human blood: a 130 kDa prodomain, a 100 kDa mature form, and a 30 kDa prodomain [40]. In addition to T-cadherin, adiponectin receptors (AdipoRs) and calreticulin have also been identified as receptors for adiponectin [41]. T-cadherin is the major binding partner of circulating adiponectin [41]. The human T-cadherin ELISA kits used in this study were cross-compatible with mouse plasma, except for the 30 kDa form. Therefore, the levels of 100 kDa and 130 kDa T-cadherin in mouse plasma were measured. As shown in Table 2, dietary NMN significantly increased plasma 100 kDa and 130 kDa T-cadherin levels, which is consistent with the increase in plasma adiponectin levels (Figure 3).

As shown in Table 2, plasma ALT and ChE activities were significantly higher and plasma albumin levels significantly lower in the NMN group than in the CON group, raising concerns regarding the safety of NMN administration. An acute toxicity study in female Sprague–Dawley rats showed that an oral lethal dose of 2666 mg/kg NMN did not result in any mortality or treatment-related adverse events [23]. In a chronic toxicity study in male and female Sprague–Dawley rats, NMN was orally administered repeatedly at doses of 375, 750, and 1500 mg/kg/d over a subchronic period of 90 d, followed by a 28 d treatment-free recovery period [23]. Although changes in several parameters, including increases in ALT activity and kidney weight, were observed after the administration of 750 and 1500 mg/kg/d of NMN for 90 d, the severity was very low, and no relevant clinicopathological changes were observed [23]. These results indicate that repeated oral administration of NMN at doses up to 1500 mg/kg/d appears to be safe and does not promote toxic effects, suggesting that the NOAEL is greater than 1500 mg/kg/d [23]. Additionally, an increase in ALT activity was observed in an acute toxicity study in which C57BL/6J mice were orally administered a high dose of NMN twice daily (2680 mg/kg/d) [42]. However, no pathological findings or clinical pathological changes were observed in the liver or kidneys, suggesting that the physiological functions of these organs were not impaired [42]. Considering that the NMN intake in this study was 902 ± 26 mg/kg/d, we believe that changes in plasma ALT and ChE activities and plasma albumin levels after dietary NMN intake are not adverse events. Several studies in human subjects have also suggested that hepatic enzyme activities may transiently increase with diet-induced weight loss [43,44,45]. *db/db* mice in the CON group exhibited hepatomegaly and obesity, whereas those in the NMN group significantly alleviated hepatomegaly and obesity (Table 1). Therefore, we hypothesize that dietary NMN-induced changes in hepatic enzyme activities are compensatory for the suppression of the development of hepatomegaly and obesity.

The present study has several limitations. First, further animal experiments and human safety evaluations (including gender effects) are needed to determine the minimum effective dose at which dietary NMN intake exerts its beneficial effects and to determine its safety. Second, we used obese diabetic *db/db* mice, which have low energy metabolism and insufficient leptin action. Further studies are needed to determine whether similar outcomes, such as the enhancement of energy expenditure with fat oxidation, can be achieved using dietary NMN intake in animal models of diet-induced obesity.

## 5. Conclusions

In summary, our results indicate that dietary NMN alleviates body fat mass and hypertriglyceridemia by enhancing energy expenditure, promoting fat oxidation, enhancing hepatic lipolysis, and suppressing hepatic lipogenesis in obese and diabetic *db/db* mice. To the best of our knowledge, this is the first report that dietary NMN intake but not oral administration via drinking water or intraperitoneal administration exerts beneficial effects such as anti-obesity and TG-lowering effects. Oral supplementation with NMN may be effective at improving obesity and obesity-associated hypertriglyceridemia in obese individuals with low energy expenditure and/or leptin resistance.

## Figures and Tables

**Table 1:** Effects of dietary NMN intake on morphometric variables in obese diabetic *db/db* mice.

	C57BL/6J	*db/db*	
	NOR Group	CON Group	NMN Group
Initial B.W. (g)	19.3 ± 0.3	27.5 ± 0.5	27.6 ± 0.6
Final B.W. (g)	24.0 ± 0.6	37.4 ± 0.9	29.7 ± 0.3 ^†^
B.W. gain (g)	4.73 ± 0.44	9.88 ± 0.80	2.14 ± 0.52 *
Food intake (g/day)	2.88 ± 0.05	5.41 ± 0.02	5.36 ± 0.14
NMN intake (mg/kg/day)	---	---	902 ± 26
Food efficiency (mg B.W. gain/g food intake)			
	58.5 ± 4.5	65.2 ± 5.2	14.3 ± 3.3 *
Water intake (mL/day)	3.88 ± 0.13	16.5 ± 0.8	23.9 ± 1.9 *
Naso–anal length (cm)	9.07 ± 0.15	9.03 ± 0.06	8.69 ± 0.05 *
Lee index	318 ± 4	370 ± 2	356 ± 1 *
Organ weight (g)			
Liver	0.985 ± 0.026	2.44 ± 0.13	1.86 ± 0.07 *
Spleen	0.0703 ± 0.0018	0.0331 ± 0.0013	0.0316 ± 0.0020
Testis	0.188 ± 0.006	0.141 ± 0.011	0.131 ± 0.011
Kidneys	0.328 ± 0.006	0.436 ± 0.009	0.453 ± 0.014
Quadriceps femoris	0.402 ± 0.029	0.185 ± 0.009	0.164 ± 0.009
WAT weight (g)			
Epididymal	0.363 ± 0.027	1.63 ± 0.12	0.959 ± 0.064 *
Perirenal	0.154 ± 0.024	0.725 ± 0.064	0.608 ± 0.027
Mesenteric	0.233 ± 0.017	1.32 ± 0.05	0.946 ± 0.019 *
Abdominal ^#^	0.750 ± 0.067	3.67 ± 0.17	2.51 ± 0.06 *
Subcutaneous	0.550 ± 0.069	4.09 ± 0.12	2.50 ± 0.06 *
BAT weight (g)	0.139 ± 0.010	0.309 ± 0.030	0.283 ± 0.028
Feces weight (g/day)	0.271 ± 0.010	0.519 ± 0.011	0.449 ± 0.040
Rectal temperature (°C)	36.6 ± 0.1	34.4 ± 0.1	35.9 ± 0.2 ^†^

BAT, brown adipose tissue; B.W., body weight; NMN, nicotinamide mononucleotide; CON group, *db/db* mice fed the control diet; NMN group, *db/db* mice fed the NMN diet; NOR group, C57BL/6J mice fed control diet; WAT, white adipose tissue. Data are expressed as the mean ± the standard error of the mean (NOR and CON groups, *n* = 6/group; NMN group, *n* = 5/group). * *p* < 0.05 (vs. CON group) analyzed using Student’s *t*-test. ^†^ *p* < 0.05 (vs. CON group) analyzed using Welch’s *t*-test. ^#^ Abdominal WAT weights were calculated by summing the weights of the epididymal, perirenal, and mesenteric WAT.

**Table 2:** Effects of dietary NMN intake on biochemical parameters in plasma, the liver, epididymal and mesenteric WAT of obese diabetic *db/db* mice.

	C57BL/6J	*db/db*	
	NOR Group	CON Group	NMN Group
Plasma biochemical parameters			
TG (mg/dL)	74.1 ± 6.5	143 ± 15	67.7 ± 13.0 *
HDL TG (mg/dL)	43.3 ± 3.0	69.9 ± 6.0	46.2 ± 5.7 *
Non-HDL TG (mg/dL)	30.7 ± 4.9	72.8 ± 10.9	21.5 ± 7.9 *
T-Chol (mg/dL)	133 ± 6	221 ± 9	262 ± 22
HDL Chol (mg/dL)	118 ± 5	202 ± 11	241 ± 19 ^(*p* = 0.096)^
Non-HDL Chol (mg/dL)	14.8 ± 3.6	19.3 ± 4.0	20.3 ± 7.6
PL (mg/dL)	243 ± 9	329 ± 16	362 ± 21
Glucose (mg/dL)	285 ± 33	611 ± 33	558 ± 40
Insulin (ng/mL)	0.0188 ± 0.0057	6.35 ± 1.43	5.90 ± 0.86
Leptin (ng/mL)	1.69 ± 0.39	61.5 ± 2.9	56.1 ± 1.9
Adiponectin (µg/mL)	26.8 ± 0.6	10.7 ± 0.4	19.6 ± 2.1 ^†^
T-Cadherin (ng/mL)			
100 kDa + 130 kDa	192 ± 9	144 ± 5	199 ± 13 *
100 kDa	166 ± 8	122 ± 4	165 ± 12 ^†^
130 kDa	26.0 ± 2.5	21.3 ± 1.0	34.0 ± 1.5 *
ALT (IU/L)	5.46 ± 0.36	24.4 ± 1.8	43.5 ± 2.8 *
ChE (IU/L)	12.9 ± 1.0	38.7 ± 1.8	55.7 ± 2.4 *
Albumin (g/dL)	3.14 ± 0.05	4.09 ± 0.06	2.99 ± 0.05 *
Hepatic biochemical parameters			
TG (mg/liver) T-Chol (mg/liver)	27.8 ± 3.2	408 ± 65	269 ± 24 ^(*p* = 0.097)^
	6.43 ± 0.69	42.0 ± 3.1	38.4 ± 0.9
F-Chol (mg/liver)	3.24 ± 0.16	9.98 ± 0.44	9.15 ± 0.37
Chol-E (mg/liver)	3.18 ± 0.59	32.0 ± 2.7	29.2 ± 1.1
PL (mg/liver)	29.4 ± 0.9	59.5 ± 2.3	53.5 ± 1.8 ^(*p* = 0.082)^
Glycogen (mg/liver)	3.26 ± 1.36	42.2 ± 7.6	20.1 ± 7.7 ^(*p* = 0.074)^
NAD^+^ (nmol/liver)	11.0 ± 1.8	21.5 ± 1.7	227 ± 48 ^†^
NADH (nmol/liver)	15.7 ± 1.2	33.4 ± 4.3	660 ± 145 ^†^
Epididymal WAT			
NAD^+^ (nmol/Epi WAT)	4.02 ± 0.54	15.8 ± 2.4	7.50 ± 2.11 *
NADH (nmol/Epi WAT)	0.287 ± 0.015	0.748 ± 0.051	0.510 ± 0.043 *
Mesenteric WAT			
Adiponectin	59.2 ± 4.6	61.3 ± 10.5	70.2 ± 10.8
(mg/g protein)			

ALT, alanine aminotransferase; ChE, cholinesterase; Chol-E, cholesterol esters; Epi, epididymal; F-Chol, free cholesterol; HDL, high-density lipoprotein; NAD^+^, nicotinamide adenine dinucleotide; NADH, nicotinamide adenine dinucleotide hydride; PL, phospholipids; T-Chol, total cholesterol; TG, triglyceride; WAT, white adipose tissue. Data are expressed as the mean ± standard error of the mean (NOR and CON groups, *n* = 6/group; NMN group, *n* = 5/group). * *p* < 0.05 (vs. CON group) analyzed using Student’s *t*-test. ^†^ *p* < 0.05 (vs. CON group) analyzed using Welch’s *t*-test.

**Table 3:** Effects of dietary NMN intake on mRNA levels in the liver and epididymal WAT of obese dieabetic *db/db* mice.

	C57BL/6J	*db/db*	
	NOR Group	CON Group	NMN Group
Liver	(Arbitrary unit)		
* Genes related to fatty acid synthesis*			
* Acaca*	56.6 ± 6.6	100 ± 9	53.5 ± 4.0 *
* Fasn*	30.8 ± 4.1	100 ± 26	35.3 ± 8.9 ^(*p* = 0.055)^
* Srebf1*	105 ± 16	100 ± 11	143 ± 17 ^(*p* = 0.060)^
* Nr1h3*	110 ± 18	100 ± 7	87.6 ± 9.0
* Genes related to fatty acid* *β-oxidation*			
* Cpt1a*	117 ± 18	100 ± 7	87.6 ± 7.0
* Cpt2*	102 ± 11	100 ± 6	123 ± 8 *
* Genes related to NAD^+^ metabolism*			
* Nmnat1*	89.3 ± 10.2	100 ± 7	86.7 ± 8.7
* Sirt1*	143 ± 8	100 ± 4	104 ± 6
* Genes related to adiponectin signaling*			
* Adipor1*	93.2 ± 10.9	100 ± 7	88.4 ± 6.7
* Adipor2*	160 ± 10	100 ± 8	63.6 ± 3.3 *
Epididymal WAT			
* Gene related to fatty acid synthesis*			
* Fasn*	91.5 ± 25.4	100 ± 27	66.9 ± 20.1
* Gene related to adipocytokine*			
* Adipoq*	133 ± 13	100 ± 15	99.3 ± 21.4
* Genes related to thermogenesis*			
* Ucp2*	36.8 ± 6.4	100 ± 18	68.0 ± 9.0
* Adrb3*	425 ± 92	100 ± 27	78.6 ± 14.7
* Prdm16*	262 ± 52	100 ± 15	113 ± 42

CON group, *db/db* mice fed the control diet; NMN group, *db/db* mice fed the NMN diet; NOR group, C57BL/6J mice fed the control diet; WAT, white adipose tissue. Data are expressed as the mean ± standard error of the mean (NOR and CON groups, *n* = 6/group; NMN group, *n* = 5/group). * *p* < 0.05 (vs. CON group) analyzed using Student’s *t*-test.

## Data Availability

The data presented in this study are available upon request from the corresponding author. These data are not publicly available because of the lack of a public archive platform for data sharing.

## Supplementary Materials

The following supporting information can be downloaded from https://www.mdpi.com/article/10.3390/metabo15050333/s1. Figure S1: Selection of the best housekeeping gene in the liver and epididymal WAT using BestKeeper; Figure S2: NAD^+^ metabolism; Table S1: Composition of the experimental diets used in this study; Table S2: Primer sequences of housekeeping and target genes in the liver and epididymal WAT used for RT-qPCR.

## Abbreviations

The following abbreviations are used in this manuscript:

ALT	Alanine aminotransferase
BAT	Brown adipose tissue
ChE	Cholinesterase
Chol-E	Cholesterol esters
CPT	Carnitine palmitoyltransferase
FAS	Fatty acid synthase
F-Chol	Free cholesterol
HDL	High-density lipoprotein
NAD^+^	Nicotinamide adenine dinucleotide
NADH	Nicotinamide adenine dinucleotide hydride
NMN	Nicotinamide mononucleotide
NOAEL	No-observable adverse effect level
PL	Phospholipids
RQ	Respiratory quotient
TG	Triglycerides
T-Chol	Total cholesterol
WAT	White adipose tissue
