# Supplementary material for: Dietary Nicotinamide Mononucleotide, a Key NAD+ Intermediate, Alleviates Body Fat Mass and Hypertriglyceridemia by Enhancing Energy Expenditure with Promotion of Fat Oxidation and Hepatic Lipolysis and Suppressing Hepatic Lipogenesis in db/db Mice
Source: Metabolites. 2025 May 18;15(5):333. doi: 10.3390/metabo15050333 (PMC12113554; doi:10.3390/metabo15050333)
Supplement: Supplementary file 1 [file metabolites-15-00333-s001.zip › metabolites-3460866-supplementary.pdf]

Dietary nicotinamide mononucleotide, a key NAD^+ intermediate, alleviates body fat mass and hypertriglyceridemia by enhancing energy expenditure with promotion of fat oxidation and hepatic lipolysis and suppressing hepatic lipogenesis in *db/db* mice

Bungo Shirouchi [1,2,*], Sarasa Mitsuta [2], Mina Higuchi [2], Mai Okumura [2], and Kazunari Tanaka [3]

[1] Department of Nutrition Science, Faculty of Nursing and Nutrition, University of Nagasaki, Siebold, 1-1-1 Manabino, Nagayo-cho, Nishi-Sonogi-gun, Nagasaki 851-2195, Japan

[2] Nutrition Science Course, Division of Human Health Science, Graduate School of Regional Design and Creation, University of Nagasaki, Siebold, 1-1-1 Manabino, Nagayo-cho, Nishi-Sonogi-gun, Nagasaki 851-2195, Japan

[3] Regional Partnership Center, University of Nagasaki, Siebold, 1-1-1 Manabino, Nagayo-cho, Nishi-Sonogi-gun, Nagasaki 851-2195, Japan

[*]Correspondence: bshirouchi@sun.ac.jp; Tel/fax: +81-95-813-5734

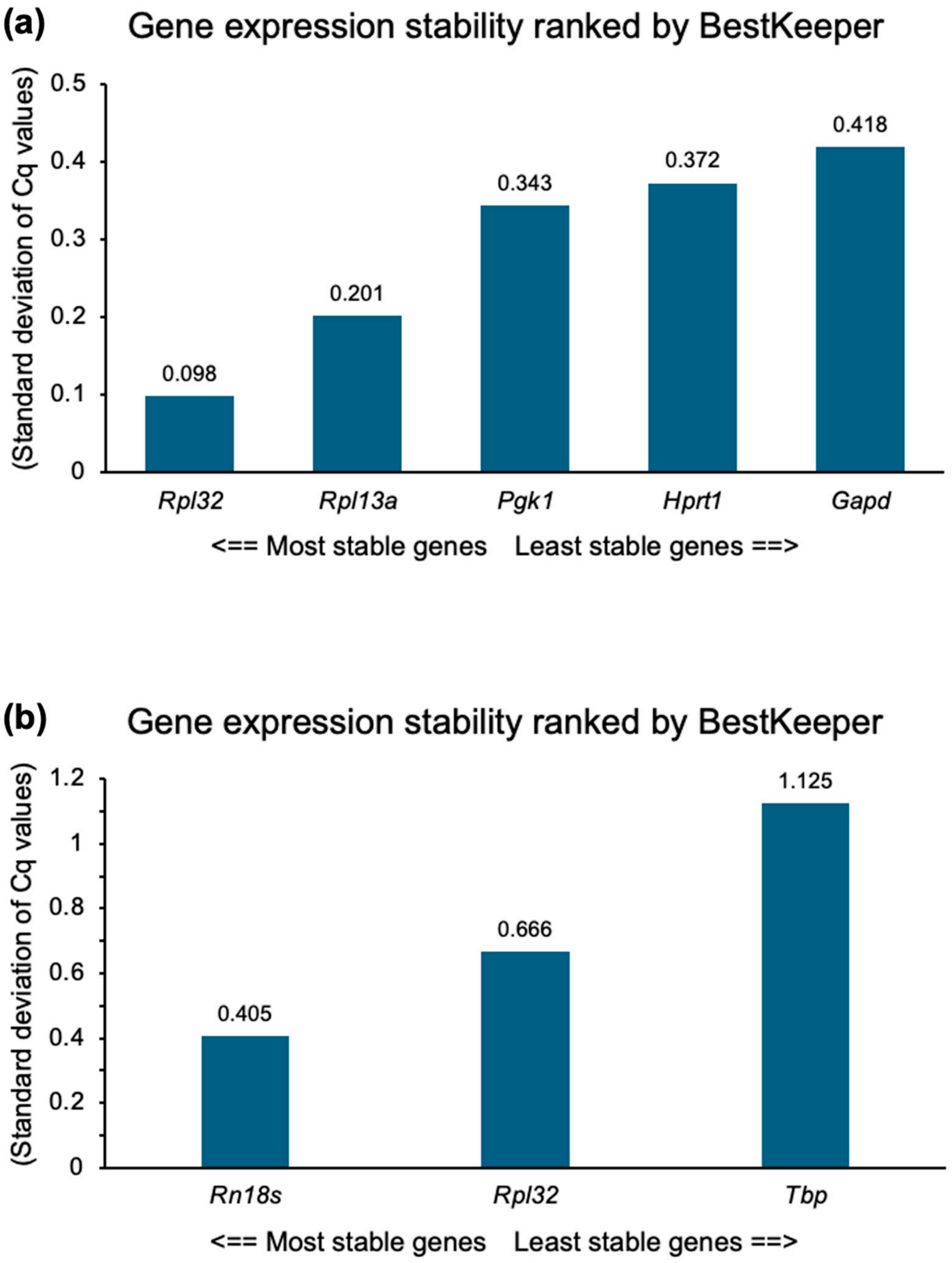

Figure S1. Selection of the best housekeeping gene in (a) the liver and (b) epididymal WAT using BestKeeper. BestKeeper calculates the stability of the candidate genes based on the standard deviation (SD) of their quantification cycle (Cq) values. According to the results, *Rpl32* in the liver and *Rn18s* in epididymal WAT were selected as the most robust housekeeping gene, respectively.

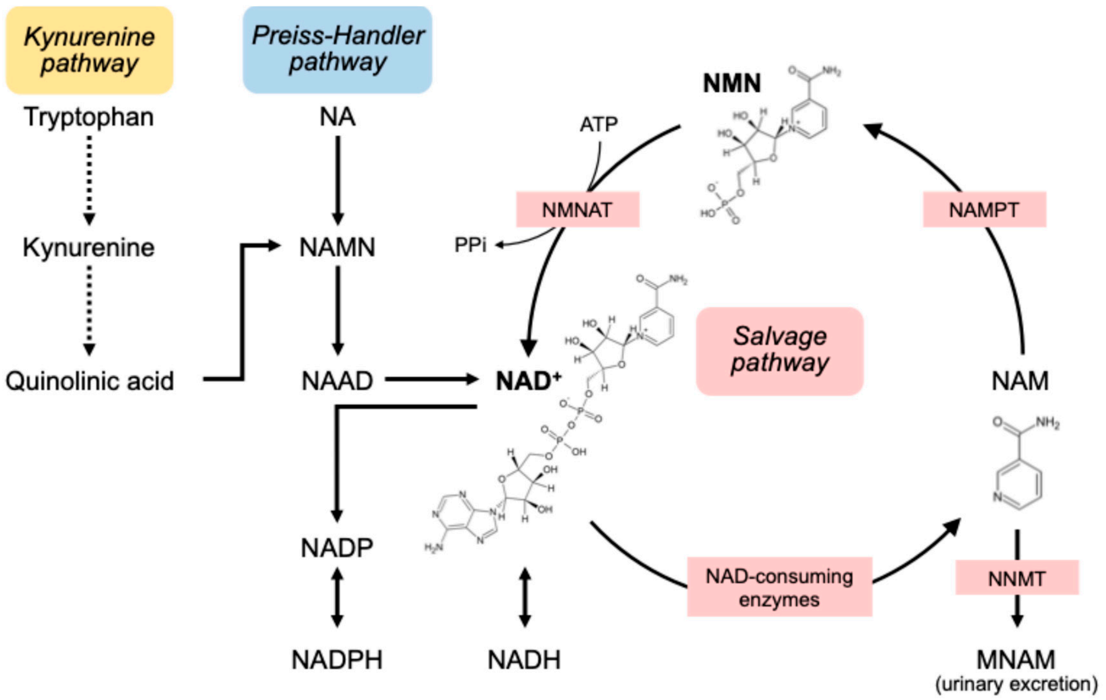

Figure S2. NAD^+ metabolism. NA, nicotinic acid; NAMN, nicotinic acid mononucleotide; NAAD, nicotinic acid adenine dinucleotide; NAM, nicotinamide; MNAM, N^1-methylnicotinamide; NAD^+, nicotinamide adenine dinucleotide; NADH, nicotinamide adenine dinucleotide hydride; NADP, nicotinamide adenine dinucleotide phosphate; NADPH, nicotinamide adenine dinucleotide phosphate hydride; NMN, nicotinamide mononucleotide; NMNAT, nicotinamide mononucleotide adenylyltransferase; NAMPT, nicotinamide phosphoribosyltransferase; NNMT, nicotinamide *N*-methyltransferase.

Table S1. Composition of the experimental diets used in this study

	Control diet	NMN diet
Ingredients	(g/kg diet)	
Sucrose	479	474
Casein	200	200
β-Cornstarch	150	150
Cellulose	50	50
Corn oil	70	70
NMN	---	5
Mineral mixture (AIN-76)	35	35
Vitamin mixture (AIN-76)	10	10
DL-Methionine	3	3
Choline bitartrate	2	2
Cholesterol	1	1

Table S2. Primer sequence of housekeeping and target genes used for real-time PCR in the present study.

Gene name[#]	Accession no.	Product size	Direction	Primer sequence (5'→3')
Liver				
Rpl32	NM_172086.2	63	F	GAAACTGGCGGAAACCCA
			R	GGATCTGGCCCTTGAACCTTC
Rpl13a	NM_009438	120	F	CTGCTCTCAAGGTTGTTCGGCT
			R	CCTTCCGTTTCTCCTCCAGAGT
Gapd	NM_008084	153	F	CATCACTGCCACCCAGAAGACTG
			R	ATGCCAGTGAGCTTCCCGTTCAG
Pgk1	NM_008828	117	F	GATGCTTTCCGAGCCTCACTGT
			R	ACCAGCCTTCTGTGGCAGATTC
Hprt1	NM_013556	146	F	CTGGTGAAAAGGACCTCTCGAAG
			R	CCAGTTTCACTAATGACACAAACG
Fasn	NM_007988.3	137	F	GGTTACACTGTGCTAGGTGTTG
			R	TCCAGGCGCATGAGGCTCAGC
Acaca	NM_133360.3	232	F	CTTCCTGACAAACGAGTCTGG
			R	CTGCCGAAACATCTCTGGGA
Nr1h3	NM_013839.4	150	F	CTCAATGCCTGATGTTTCTCCT
			R	TCCAACCCTATCCCTAAAGCAA
Cpt1a	NM_013495.2	169	F	ATCTGGATGGCTATGGTCAAGGTC
			R	GTGCTGTCATGCGTTGGAAGTC
Cpt2	NM_009949.2	117	F	GCCTGCTGTTGCGTGACTG
			R	TGGTGGGTACGATGCTGTGC
Sirt1	NM_019812.3	86	F	GGAACCTTTGCCTCATCTACA
			R	CACCTAGCCTATGACACAACTC
Nmnat1	NM_133435.2	131	F	TGGCTCTTTTAACCCCATCAC
			R	TCTTCTTGTACGCATCACCGA
Adipor1	NM_028320.4	133	F	ACGTTGGAGAGTCATCCCGTAT
			R	CTCTGTGTGGATGCGGAAGAT
Adipor2	NM_197985.4	140	F	GGAGTGTTCGTGGGCTTAGG
			R	GCAGCTCCGGTGATATAGAGG

[#]Gene names are represented by approved symbols. F, forward; R, reverse.

Rpl32, ribosomal protein L32; *Rpl13a*, ribosomal protein L13a; *Gapd*, glyceraldehyde-3-phosphate dehydrogenase; *Pgk1*, phosphoglycerate kinase 1; *Hprt1*, hypoxanthine phosphoribosyltransferase 1; *Fasn,* fatty acid synthase; *Acaca*, acetyl-Coenzyme A carboxylase alpha; *Nr1h3*, nuclear receptor subfamily 1, group H, member 3; *Cpt1a*, carnitine palmitoyltransferase 1a; *Cpt2*, carnitine palmitoyltransferase 2; *Sirt1*, sirtuin 1; *Nmnat1*, nicotinamide nucleotide adenylyltransferase 1; *Adipor1*, adiponectin receptor 1; *Adipor2*, adiponectin receptor 2.

Table S2. Primer sequence of housekeeping and target genes used for real-time PCR in the present study (*continued*).

Gene name#	Accession no.	Product size	Direction	Primer sequence (5'→3')
Epididymal WAT				
Rpl32	NM_172086.2	63	F	GAAACTGGCGGAAACCCA
			R	GGATCTGGCCCTTGAACCTTC
Tbp	NM_013684.3	129	F	GAAGAACAATCCAGACTAGCAGCA
			R	CCTTATAGGGAACTTCACATCACAG
Fasn	NM_007988.3	137	F	GGTTACACTGTGCTAGGTGTTG
			R	TCCAGGCGCATGAGGCTCAGC
Adipsin	NM_013459.4	129	F	CATGCTCGGCCCTACATGG
			R	CACAGAGTCGTCATCCGTCAC
Ucp2	NM_011671.6	126	F	TAGTGCGCACCGCAGCC
			R	AGCTCATCTGGCGCTGCAG
Adrb3	NM_013462.3	121	F	GGCAACCTGCTGGTAATCAT
			R	CTGGTGGCATTACGAGGAGT

#Gene names are represented by approved symbols. F, forward; R, reverse; WAT, white adipose tissue. *Rpl32*, Ribosomal protein L32; *Tbp*, TATA box binding protein; *Fasn*, Fatty acid synthase; *Ucp2*, uncoupling protein 2 (mitochondrial, proton carrier); *Adrb3*, adrenergic receptor, beta 3.
